# Supplementary material for: Comparison of the Chemical Components, Efficacy and Mechanisms of Action of Chrysanthemum morifolium Flower and Its Wild Relative Chrysanthemum indicum Flower against Liver-Fire Hyperactivity Syndrome of Hypertension via Integrative Analyses
Source: Int J Mol Sci. 2022 Nov 9;23(22):13767. doi: 10.3390/ijms232213767 (PMC9692626; doi:10.3390/ijms232213767)
Supplement: Supplementary file 1 [file ijms-23-13767-s001.zip › ijms-1981423-supplementary.pdf]

Supplementary Methods

S1.1 Solution preparation

S1.1.1 Preparation of Modeling reagents

Zingiber officinale Roscoe (50 g), *Cinnamomum cassia* (L.) J.Presl (50 g), concocted *Aconitum carmichaelii* Debeaux (50 g) were boiled together in 1500 mL of distilled water for 30 min. After filtration, the residue was boiled again in 1500 mL of distilled water for 30 min prior to another filtration. The extracts were concentrated under vacuum to a final concentration of 1 g/mL (equal to raw medicinal materials). Subsequently, L-NAME was added to the above extraction solution, and finally, a modeling solution containing 1 g/mL raw medicinal materials and 5 mg/mL L-NAME was prepared.

S1.1.2 Preparation of administration solution

CMF or CIF (135 g) were boiled in 1350 mL of distilled water for 1.5 h, respectively. After filtration, the residue was boiled again in 1350 mL of distilled water for 1.5 h prior to another filtration. The extracts of CMF and CIF were respectively diluted with distilled water and finally prepared into extracts with a concentration of 135 mg/mL, 270 mg/mL and 540 mg/mL (equal to raw medicinal materials), respectively.

Tianma Gouteng granule (13.5 g) were dissolved in 100 mL of distilled water, and fully dissolved by ultrasonication for 10 min to prepare a positive control solution with a concentration of 135 mg/mL.

S1.2 The LC and MS conditions for HPLC-Q-TOF-MS identification and plasma metabolomics

The experiments were performed on an Agilent 1260 infinity liquid chromatography system (Agilent, Santa Clara, CA, USA) in tandem with a triple TOF 5600+ quadrupole time-of-flight tandem mass spectrometer (Sciex, Redwood City, CA, USA) via an electrospray ionization (ESI) interface.

The sample was separated on an Agilent poroshell 120 SB-AQ column (100 mm × 4.6 mm, 2.7 μm) at 30 °C. The mobile phase consists of water-formic acid (100:0.1, v/v, A) and acetonitrile-formic acid (100:0.1, v/v, B). For HPLC-Q-TOF-MS identification, the gradient program was optimized as follows: 0-10 min, 5-10% B; 10-15 min, 10-12% B; 15-25 min, 12-25% B; 25-35 min, 25-30% B; 35-40 min, 30-45% B, 40-60 min, 45-90% B. The flow rate was 0.4 mL/min, and the injection volume was 4 μL. For plasma metabolomics, The gradient program was optimized as follows: 0-5 min, 2-10% B; 5-10 min, 10-40% B; 10-20 min, 40-60% B; 20-30 min, 60-90% B. The flow rate was 0.5 mL /min, and the injection volume was 4 μL.

The optimized parameters for the MS conditions of HPLC-Q-TOF-MS identification were as

follows: ion spray voltage, -4500 V (-) / 5500V (+); the turbo spray temperature, 550 ºC; nebulizer gas (nitrogen), 55 psi; heater gas (nitrogen), 55 psi; curtain gas (nitrogen), 35 psi. In the TOF-MS mode, scan range, *m*/*z* 100-1500 Da; declustering potential (DP), -60 V (-)/60 V (+); collision energy (CE), -10 V (-)/ 10 V (+), in the TOF MS/MS mode, DP, -60 V (-) / 60 V (+); CE, -35V (-)/ 35 V (+); scan range, *m*/*z* 100-1000 Da. For plasma metabolomics, the optimized parameters for the MS conditions were as follows: ion spray voltage, -4500 V (-) / 5500V (+); the turbo spray temperature, 550 ºC; nebulizer gas (nitrogen), 50 psi; heater gas (nitrogen), 50 psi; curtain gas (nitrogen), 30 psi. In the TOF-MS mode, scan range, *m*/*z* 50-1200 Da; DP, -80 V (-)/80 V (+); CE, -10 V (-)/ 10 V (+), in the TOF MS/MS mode, DP, -80 V (-) / 80 V (+); CE: -35V (-)/ 35 V (+), scan range, *m*/*z* 50-1200 Da.

S1.3 Analytical method validation for plasma metabolomics

In order to ensure the accuracy and reliability of the established method, the method was verified. The precision of the instrument was verified by 6 consecutive injections of the same quality control (QC) sample. To investigate the method repeatability, 6 independently processed parallel samples were prepared and analyzed. The stability of the samples was examined by injection of the same QC sample separately at 0, 4, 6, 8, 12 h and 24 h.

Supplementary Tables

Table S1. Information of the 91 *in vitro* chemical constituents identified in *Chrysanthemum morifolium* flower (CMF) and *Chrysanthemum indicum* flower (CIF).

No	t_R (min)	Name	Formula	Ion adduction	Found at Mass (Da)	Fragments	Error (ppm)	CMF	CIF
				**Flavonoids and their analogues**					
1	43.17	Acacetin	$C_{16}H_{12}O_5$	$[M-H]^-$	283.0611	283.0573, 268.0378, 240.0443	-0.3	√	√
2	43.63	Genkwanin	$C_{16}H_{12}O_5$	$[M-H]^-$	283.06093	281.8447, 268.0378, 240.0443, 214.9495, 211.0401, 191.9448, 174.9552,	-0.9	√	√
3	39.85	Prunetin	$C_{16}H_{12}O_5$	$[M-H]^-$	283.06109	269.0413, 239.0329, 211.0331, 195.0437, 179.0339, 161.0250, 151.0030	-0.4	√	√
4	39.85	Acacetin-7-O-glucoside	$C_{22}H_{22}O_{10}$	$[M-H]^-$	445.1134	445.1122, 401.0809, 357.0968, 330.8814 283.0578, 225.0573, 221.0453	-1.4	√	
5	40.51	Acacetin-7-O-glucuronside	$C_{22}H_{20}O_{11}$	$[M-H]^-$	459.09293	459.0900, 283.0600.268.0369, 175.0259, 117.0197, 113.0250, 85.0315	-0.8	√	√
6	43.17	Acacetin-7-O-6'-malonylgalactoside	$C_{25}H_{24}O_{13}$	$[M-H]^-$	531.11435	283.0607, 268.0369	-0.1	√	√
7	36.7	Apigenin	$C_{15}H_{10}O_5$	$[M-H]^-$	269.04537	227.0315, 197.0627, 161.0228, 159.0481, 151.0070	-0.7	√	
8	36.43	Sulfuretin	$C_{15}H_{10}O_5$	$[M-H]^-$	269.04537	269.0222, 268.0325, 214.9251, 159.0452, 151.0062, 117.0376	-0.4	√	√
9	32.81	Apigenin-7-O-glucoside	$C_{21}H_{20}O_{10}$	$[M-H]^-$	431.09771	431.0950, 268.0365, 269.0447	-1.5	√	√
10	33.24	Apigenin-7-O-glucuronside	$C_{21}H_{18}O_{11}$	$[M-H]^-$	445.0768	445.0753, 269.0456, 113.0344, 85.0317	-1.9	√	√
11	36.7	Apigenin-7-O-6'-acetylglucoside	$C_{23}H_{22}O_{11}$	$[M-H]^-$	473.10832	473.1064, 269.0438, 268.0342	-1.3	√	√
12	44.75	Apigenin-7-O-6'-malonylglucoside	$C_{24}H_{22}O_{13}$	$[M-H]^-$	517.09819	473.1102, 269.0446	-1.1	√	√
13	31.45	Apigenin-7-O-rutinoside	$C_{27}H_{30}O_{14}$	$[M-H]^-$	577.15592	577.1533, 269.0458	-1	√	√
14	44.43	Diosmetin	$C_{16}H_{12}O_6$	$[M-H]^-$	299.05558	299.0556,284.0323,256.0387	-1.8	√	√
15	37.59	Diosmetin-isomer	$C_{16}H_{12}O_6$	$[M-H]^-$	299.05548	299.0520, 284.0263, 255.0332, 184.8791, 173.9936, 166.8711, 119.9491	-2.1	√	
16	42.12	Chrysoeriol	$C_{16}H_{12}O_6$	$[M-H]^-$	299.05517	284.0334	-3.2	√	√
17	37.56	Diosmetin-7-O-6'-acetylglucoside	$C_{24}H_{24}O_{12}$	$[M-H]^-$	503.11926	299.0540, 284.0353	-0.5	√	
18	37.56	Diosmetin-7-O-6'-malonylglucoside	$C_{25}H_{24}O_{14}$	$[M-H]^-$	547.10889	503.1190, 299.0559, 284.0319	-0.8	√	√
19	33.84	Diosmetin-7-O-glucoside	$C_{22}H_{22}O_{11}$	$[M-H]^-$	461.1087	461.2401, 415.2239, 299.0548, 284.0320, 283.0334, 211.8976, 161.0275	-0.5	√	√
20	34.18	Diosmetin-7-O-glucuronside	$C_{22}H_{20}O_{12}$	$[M-H]^-$	475.08783	475.0881, 299.0553, 284.0316, 175.0224, 117.0211, 113.0253, 85.0294	-0.8	√	√
21	40.22	Luteolin	$C_{15}H_{10}O_6$	$[M-H]^-$	285.04027	199.0402, 175.0390, 151.0075, 133.0303	-0.7	√	√
22	30.39	Luteolin-7-O-glucoside	$C_{21}H_{20}O_{11}$	$[M-H]^-$	447.09287	385.1421, 285.0387, 284.0309, 243.1227, 240.9235, 201.0415, 174.9563	-0.9	√	√

23	30.59	Luteolin-7-O-glucuronside	$C_{21}H_{18}O_{12}$	$[M-H]^-$	461.07236	461.0703, 447.0972, 307.0245, 285.0398,	-0.4	√	√
24	37.02	Luteolin-7-O-6'-acetylglucoside	$C_{23}H_{22}O_{12}$	$[M-H]^-$	489.10401	489.1003, 285.0387, 284.0310	0.3	√	√
25	33.3	Luteolin-7-O-6'-malonylglucoside	$C_{24}H_{22}O_{14}$	$[M-H]^-$	533.09345	489.1013, 285.0391, 284.0316	-0.4	√	√
26	29.53	Luteolin-7-O-rutinoside	$C_{27}H_{30}O_{15}$	$[M-H]^-$	593.15073	285.039, 170.9513, 165.0527, 148.9257, 120.0263, 112.9847, 102.9571	-0.8	√	√
27	25.38	Kaempferol-3-rutinoside	$C_{27}H_{30}O_{15}$	$[M-H]^-$	593.15096	503.1181, 473.1069, 445.0875, 417.0736, 396.0900, 383.0775, 375.0692	-0.4	√	√
28	35.07	Naringenin-7-O-6'-acetylglucoside	$C_{23}H_{24}O_{11}$	$[M-H]^-$	475.12438	412.1771, 360.9090, 292.9011, 272.0577, 177.0151, 151.0026, 146.9660	-0.4	√	
29	31.33	Naringenin	$C_{15}H_{12}O_5$	$[M-H]^-$	273.07594	271.0580, 177.0184, 165.0182, 151.0019, 119.0495, 107.0131, 93.0348	0.2	√	
30	31.33	Naringenin-7-O-glucoside	$C_{21}H_{22}O_{10}$	$[M-H]^-$	433.11371	433.1031, 271.0505	-1	√	√
31	31.93	Hesperetin-7-O-glucuronside	$C_{22}H_{22}O_{12}$	$[M-H]^-$	477.10424	314.0429, 301.0527, 299.0183, 286.0332, 285.0394, 271.0226, 257.0385	0	√	√
32	28.47	Eriodictyol	$C_{15}H_{12}O_6$	$[M-H]^-$	287.0568	175.0053, 151.0062, 135.0455, 107.0179	-0.7	√	√
33	28.47	Eriodictyol-7-O-glucoside	$C_{21}H_{22}O_{11}$	$[M-H]^-$	449.10827	287.0548, 175.0035, 151.0035, 135.0449	-0.8	√	√
34	28.84	Eriodictyol-7-O-glucuronside	$C_{21}H_{20}O_{12}$	$[M-H]^-$	463.08775	463.0839, 287.0549, 175.0244, 151.0040, 135.0457, 113.0246, 85.0308	-0.9	√	√
35	29.74	Quercetin-7-O-glucoside	$C_{21}H_{20}O_{12}$	$[M-H]^-$	463.08713	463.0872, 427.1560, 301.0345, 287.0557, 247.0946, 151.0035, 135.0458	-0.9	√	√
36	31.86	Eriodictyol-7-O-6'-malonylglucoside	$C_{24}H_{24}O_{14}$	$[M-H]^-$	535.10887	491.1178, 287.0555, 175.0034, 151.0037, 135.0453	-0.8	√	√
37	27.73	Eriodictyol-7-O-rutinoside	$C_{27}H_{32}O_{15}$	$[M-H]^-$	595.16626	459.1142, 288.0583, 287.0550, 269.0430, 193.0197, 151.0040, 135.0446	-1	√	√
38	27.05	Taxifolin-7-O-glucoside	$C_{21}H_{22}O_{12}$	$[M-H]^-$	465.10287	465.1027, 303.0526, 285.0421	-2.1		√
39	16.73	Taxifolin-7-O-glucuronside	$C_{21}H_{20}O_{13}$	$[M-H]^-$	479.0823	303.0491, 285.0403, 259.0561, 241.0515, 217.0533, 178.9963, 175.0403	-1	√	√
40	38.71	Quercetin	$C_{15}H_{10}O_7$	$[M-H]^-$	301.03513	301.0342,178.9991,151.0038,121.0310,107.0152	-1.7	√	√
41	30.62	Kaempferol	$C_{15}H_{10}O_6$	$[M-H]^-$	285.04022	257.0475, 243.0279, 217.0528, 199.0395, 185.0590, 171.0465, 151.0086	-0.8	√	√
42	32.74	Baicalin	$C_{21}H_{18}O_{11}$	$[M-H]^-$	445.0776	269.0444, 113.0248	-2	√	√
43	43.15	Acacetin-7-O-(6''-O-acetyl)-β-D-glucopyranoside	$C_{24}H_{24}O_{11}$	$[M-H]^-$	487.1234	283.0601, 268.0361, 254.9132, 146.9664	-2.4	√	√
44	28.51	Isorhamnetin-3,7-O-diglucoside	$C_{28}H_{32}O_{17}$	$[M-H]^-$	639.15612	315.0536	-1	√	√
45	33.4	5,8,4'-Trihydroxy-7-methoxyflavone 8-O-glucoside	$C_{22}H_{22}O_{11}$	$[M-H]^-$	461.10894	299.0550, 284.0327	-0.6	√	√
46	29.73	Quercetin-3-O-glucoside	$C_{21}H_{20}O_{12}$	$[M-H]^-$	463.08793	301.0347, 300.0264, 271.0289, 255.0278, 178.9987, 151.0060	-0.6	√	√
47	32.48	Diosmetin7-O-galactoside	$C_{23}H_{26}O_{11}$	$[M-H]^-$	477.13955	301.0696	-1.4		√
48	36.56	Acacetin-7-O-galactoside	$C_{22}H_{22}O_{10}$	$[M-H]^-$	445.11418	445.2942, 283.0604, 268.0414, 194.9040, 104.9541	-3.3	√	
49	27.92	6-C-xylosyl-8-C-glucosylapigenin	$C_{26}H_{28}O_{14}$	$[M+H]^+$	565.1549	547.1445, 529.1385, 481.0997，469.1110, 409.0916, 379.0731, 349.0715	-0.3	√	√
50	25.85	6-C-arabinosyl-8-C-glucosylapigenin	$C_{26}H_{28}O_{14}$	$[M+H]^+$	565.15561	529.1306, 511.1248, 475.1201, 427.1029, 409.0941, 391.0783, 379.0832	-2.5	√	√
51	26.65	6-C-glucosyl-8-C-arabinosylapigenin	$C_{26}H_{28}O_{14}$	$[M+H]^+$	565.15518	547.1378, 529.1347, 511.1191, 493.0911, 427.1061, 409.0957, 379.0959	-1.3	√	√
52	27.16	6-C-β-D-Glucopyranosyl-8-C-β-D-xylopyranosylapige	$C_{26}H_{28}O_{14}$	$[M+H]^+$	565.15541	547.1378，529.134,　493.1005, 427.1061, 409.0975, 379.0959, 351.0707	0.6	√	√

		nin							
53	27.97	5,3′,4′-Tetrahydroxy-dihydroflavone-7-O-glucoside	$C_{21}H_{22}O_{11}$	$[M+H]^+$	451.1238	289.0695,163.0376,153.0173	0.7	√	√
54	28.83	Rutin	$C_{27}H_{30}O_{16}$	$[M-H]^+$	609.14591	300.0281, 271.0227	0.1	√	√
55	28.44	5,7,3',4'-Tetrahydroxyflavonone	$C_{15}H_{12}O_6$	$[M-H]^-$	287.05596	172.9741, 151.0053, 135.0416, 134.0347, 125.0252,	-1.5	√	√
56	36.88	Linarin	$C_{28}H_{32}O_{14}$	$[M-H]^-$	591.17169	283.0593，268.0379	-0.2	√	√
				**Organic acid and their analogues**					
57	20.43	Caffeic acid	$C_9H_8O_4$	$[M-H]^-$	179.03508	135.0459,134.03880	-0.2	√	√
58	26.14	p-coumaric acid	$C_9H_8O_3$	$[M-H]^-$	163.04031	119.0510,117.0435,94.9297,93.0350	3.7	√	√
59	4.3	Succinic acid	$C_4H_6O_4$	$[M-H]^-$	117.01989	73.0308	4.8	√	√
60	13.28	4-hydroxy-benzoic acid	$C_7H_6O_3$	$[M-H]^-$	137.02479	137.0241,119.0131,109.0304,108.0226,92.0280,81.0364,53.0433	1.9	√	√
61	3.13	Quinic acid	$C_7H_{12}O_6$	$[M-H]^-$	191.05626	191.0542,173.0441,127.0393,109.0301,93.0350,85.0304,59.0168	-0.1	√	√
62	12.48	4-O-Caffeoylquinic acid	$C_{16}H_{18}O_9$	$[M-H]^-$	353.08723	191.0057,179.0346,135.0454	-2	√	√
63	19.38	1-O-Caffeoylquinic acid	$C_{16}H_{18}O_9$	$[M-H]^-$	353.0876	191.0560,179.0350,173.0463,135.0354	-1.5	√	√
64	20.35	5-O-Caffeoylquinic acid	$C_{16}H_{18}O_9$	$[M-H]^-$	353.08767	191.0561	-0.8	√	√
65	32.03	3-O-Caffeoylquinic acid	$C_{16}H_{18}O_9$	$[M-H]^-$	353.08786	191.0569, 179.0365, 173.0464, 135.0472, 93.0345	0.4	√	√
66	25.94	1,5-dicaffeoylquinic acid	$C_{25}H_{24}O_{12}$	$[M-H]^-$	515.11871	515.1170,353.0865,335.0740,191.0560,179.0350,135.0460	-0.9	√	√
67	30.94	3,4-dicaffeoylquinic acid	$C_{25}H_{24}O_{12}$	$[M-H]^-$	515.11923	515.1155,353.0864,335.0773,191.0565,179.0351,173.0456,135.0458	-0.8	√	√
68	32.03	3,5-dicaffeoylquinic acid	$C_{25}H_{24}O_{12}$	$[M-H]^-$	515.11932	353.0850,191.0550,179.0343,135.0452	-0.8	√	√
69	32.96	4,5-dicaffeoylquinic acid	$C_{25}H_{24}O_{12}$	$[M-H]^-$	515.11903	353.0854, 191.0564, 179.0351, 173.0456, 135.0466	-0.9	√	√
70	24.94	p-coumaroylquinic acid	$C_{16}H_{18}O_8$	$[M-H]^-$	337.09284	191.0704,163.0393,93.0341	-0.8	√	√
71	25.34	Feruloylquinic acid	$C_{17}H_{20}O_9$	$[M-H]^-$	367.10337	367.0982,193.0483,173.0431,161.0243,137.0258,93.0383，	-1.9	√	√
72	26.25	CQA methyl ester	$C_{17}H_{20}O_9$	$[M-H]^-$	367.10321	367.1279,193.0486,191.0533,173.0464,93.0341	-0.9	√	√
73	26.69	CQA methyl ester	$C_{17}H_{20}O_9$	$[M-H]^-$	367.10267	367.1001,214.9110,184.8756,179.0335,161.0262,135.0455,133.0311	-3.6	√	√
74	16.49	Methylparaben	$C_8H_8O_3$	$[M-H]^-$	151.04015	151.0401,136.0159,108.0214	4.1	√	√
75	35.93	Methyl vanillate	$C_9H_{10}O_4$	$[M-H]^-$	181.05056	181.0502,166.0274,138.0308,123.0071	-0.4	√	√
76	33.63	diCQA methyl ester	$C_{26}H_{26}O_{12}$	$[M-H]^-$	529.13359	529.1192,367.0406,299.0527	-3.5	√	√
77	14.95	Caffeic acid-glucoside	$C_{15}H_{18}O_9$	$[M-H]^-$	341.08763	281.0652,251.0536,221.0438,179.0345,161.0249,135.0451	-0.9	√	√
78	17.18	Caffeic acid-glucoside	$C_{15}H_{18}O_9$	$[M-H]^-$	341.08734	281.0616,251.0537,221.0452,179.0334,161.0230,135.0467	-1	√	√
79	18.12	CQA glucoside	$C_{22}H_{28}O_{14}$	$[M-H]^-$	515.13915	515.1345,353.0897,323.0753,191.0537,161.0232	-0.5	√	√
80	29.38	diCQA glucoside	$C_{31}H_{34}O_{17}$	$[M-H]^-$	677.17169	515.1241,353.0906,323.0786,191.0558	-0.3	√	√

81	30.64	diCQA glucoside	$C_{31}H_{34}O_{17}$	$[M-H]^-$	677.1717	677.2078,515.1394,353.0954,179.0354	-0.3	√	√
82	10.18	Protocatechuic acid	$C_7H_6O_4$	$[M-H]^-$	153.01947	153.0193, 109.0299	-0.2	√	√
83	17.86	Gentisic acid	$C_7H_6O_4$	$[M-H]^-$	153.01946	153.0218, 109.0311	2.8	√	√
84	8.51	Vanillic acid	$C_8H_8O_4$	$[M-H]^-$	167.03496	123.0470, 108.0181	-0.1	√	√
					**Amino acids**				
85	2.85	Aspartic acid	$C_4H_7NO_4$	$[M+H]^+$	134.04478	134.0993, 130.0507, 124.9986, 114.9521, 116.0337, 102.0573	0.8	√	√
86	2.88	Glutamic acid	$C_5H_9NO_4$	$[M+H]^+$	148.06037	148.0605, 130.0503, 102.0558	-0.8	√	√
87	2.54	Lysine	$C_6H_{14}N_2O_2$	$[M+H]^+$	147.11275	130.0709, 122.9239	0.4	√	√
88	2.69	L-arginine	$C_6H_{14}N_4O_2$	$[M+H]^+$	175.11882	130.0984, 116.0718	-0.7	√	√
89	5.04	L-phenylalanine	$C_9H_{11}NO_2$	$[M+H]^+$	166.0862	166.0863, 120.0806, 103.0572	-1.1	√	√
90	4.1	Tyrosine	$C_9H_{11}NO_3$	$[M+H]^+$	182.0812	165.0541, 136.0745, 119.0484, 107.0481	-0.9	√	√
					**Others**				
91	4.34	Adenosine	$C_{10}H_{13}N_5O_4$	$[M+H]^+$	268.10439	136.0613, 119.0352	1.3	√	√

Table S2. The identification results of the prototype components (P) of CMF and CIF in plasma.

ID	Name	Formula	CMF	CIF
P1	Acacetin	$C_{16}H_{12}O_5$	√	√
P2	Apigenin-7-O-glucoside	$C_{21}H_{20}O_{10}$	√	√
P3	Apigenin-7-O-glucuronside	$C_{21}H_{18}O_{11}$	√	√
P4	Diosmetin	$C_{16}H_{12}O_6$	√	√
P5	Diosmetin-7-O-glucuronside	$C_{22}H_{20}O_{12}$	√	√
P6	Eriodictyol-7-O-glucuronide	$C_{21}H_{20}O_{12}$	√	√
P7	Hesperetin-7-O-glucuronside	$C_{22}H_{22}O_{12}$	√	√
P8	Luteolin	$C_{15}H_{10}O_6$	√	√
P9	Luteolin-7-O-glucoside	$C_{21}H_{20}O_{11}$	√	√
P10	Luteolin-7-O-glucuronside	$C_{21}H_{18}O_{12}$	√	√
P11	Quercetin-3-O-glucoside	$C_{21}H_{20}O_{12}$	√	√
P12	4,5-dicaffeoylquinic acid	$C_{25}H_{24}O_{12}$	√	√
P13	Succinic acid	$C_4H_6O_4$	√	√
P14	Gentisic acid	$C_7H_6O_4$	√	√

Table S3. The identification results of the metabolites (M) of CMF and CIF in plasma.

ID	Formula	Found at Mass (Da)	Error (ppm)	t_R (min)	Fragments	CMF	CIF
					**Flavonoids and their analogues**		
M1	$C_{15}H_{10}O_9S$	364.9957	-4.4	33.33	364.9896,285.0399,284.1509,92.9286	√	
M2	$C_{15}H_6O_7$	297.0044	1.1	16.78	305.2708,297.0047,233.0425,237.9924,92.9296，	√	
M3	$C_{12}H_2O_6$	240.9786	2.9	8.53	203.0837,172.9915,132.9079,93.0365	√	√
M4	$C_{13}H_4O_6$	254.9939	1.3	15.1	154.9939	√	√
M5	$C_{16}H_{12}O_8S$	363.0168	-3.4	46.07	363.0129,283.0594,268.0345	√	
M6	$C_{15}H_{10}O_5$	269.0451	-1.7	43.66	269.0455.268.1993,225.0594,151.0050,117.0362	√	
M7	$C_{16}H_{14}O_6$	301.0708	-3.3	42.55	301.0703,285.0406,164.9293,151.0041,134.0381	√	
M8	$C_{16}H_{12}O_6$	299.0552	-3	44.04	299.0544,284.0317,256.0373,151.0043	√	
M9	$C_{15}H_{10}O_6$	285.0398	-2.3	39.36	285.0392,241.0507,133.0306	√	√
M10	$C_{15}H_{10}O_8S$	349.0007	-4.8	30.7	349.0068,303.2156,269.0449,222.8827,146.9656	√	
M11	$C_{28}H_{28}O_{18}$	651.1171	-4.9	30.17	652.1187,651.11162,475.0854,299.0556,113.0262	√	
M12	$C_{21}H_{18}O_{12}$	461.0703	-4.9	33.64	461.0686,285.0393	√	√
M13	$C_{27}H_{26}O_{18}$	637.1025	-3.4	29.11	637.0992,461.0684,285.0389	√	√
M14	$C_{21}H_{20}O_{10}$	431.0963	-4.7	32.36	533.1417,431.0942,269.0448,168.0378,240.0415,226.9671	√	√
M15	$C_8H_{15}NO_6$	220.0832	2.2	3.09	217.0298	√	√
M16	$C_8H_{14}O_7$	221.0666	-0.3	3.41	221.0141.166.9959,148.9923,130.9811,89.0265,76.9729	√	√
M17	$C_8H_{14}O_5$	189.0771	1.3	9.03	189.0776,131.0737,127.0774,115.0413,99.0830	√	
M18	$C_7H_{14}O_7$	209.067	1.4	3.03	209.0670	√	√
M19	$C_{19}H_{14}O_{11}$	417.0459	-1.1	2.64	281.0717,213.0852,145.0989	√	√
M20	$C_{18}H_{12}O_{12}$	419.0273	4	58.66	415.0331	√	
M21	$C_6H_{10}O_5$	161.0462	4.3	4.2	161.0396,103.0421,101.0259,99.0470,57.0384	√	√
M22	$C_{21}H_{18}O_8$	397.0921	-2	2.95	217.0292,215.0351	√	√
M23	$C_6H_{12}O_6$	179.0567	3.1	2.92	426.1845,107.0334,94.9298,89.0259,71.0172,56.0172	√	√
M24	$C_6H_8O_6$	175.0254	3.2	3.3	175.0245	√	√
M25	$C_6H_8O_7$	191.0198	0.7	3.44	111.0100,87.0108,85.0315,67.0223	√	√
M26	$C_{11}H_{20}N_2O_8$	307.1133	-4.6	3.16	308.9293	√	√

M27	$C_{21}H_{24}O_9$	419.133	-4.3	32.78	419.1317,401.1222,243.1019,113.0262	√	√
M28	$C_{11}H_{18}O_{11}$	325.0767	-2.9	3.4	325.0767		√
M29	$C_{21}H_{22}O_{10}$	433.112	-4.7	33.55	433.1129,257.0817,113.0264	√	√
M30	$C_{20}H_{18}O_9$	401.0862	-4.1	45.16	401.0845,313,0695,269.0800,225.0543,121.0303	√	√
M31	$C_{23}H_{22}O_{11}$	473.1109	4.2	2.91	471.1109		√
M32	$C_{21}H_{22}O_9$	417.1175	-3.9	31.03	417.1156,399.1084,241.0864,113.0261	√	√
M33	$C_6H_{12}O_7$	195.0511	0.5	3.12	157.0370,140.0109,97.0063		√
M34	$C_8H_{12}O_6$	203.0568	3.4	2.53	203.0553,156.9023,145.0991,130.9552,128.0690,68.9878	√	√
M35	$C_8H_{12}O_8$	235.0457	-1	4.09	117.0206,116.6589,99.0100,73.0326	√	√
M36	$C_{11}H_{16}O_{12}$	339.0581	3.7	7.55	281.1047,197.0370,187.0147,129.0563,115.0296	√	√
M37	$C_{19}H_{12}O_{11}$	415.0327	4.8	58.51	415.2219,369.3345,279.2315,152.9971	√	√
M38	$C_6H_{10}O_{10}S$	272.991	-4.5	3	201.1357,191,0633,145.0623,94.9268,92.92979	√	√
M39	$C_{18}H_{10}O_{12}$	417.0101	0.5	2.91	417.0101		√
M40	$C_6H_6O_7$	189.0039	-1.1	15.12	189.0048,143.1052,109.0580,107.0522,81.9556		√
M41	$C_7H_{12}O_7$	207.051	-0.3	3.13	127.0452,117.0194,101,0272,87.0112,84.0278,85.0364,61.9933		√
M42	$C_7H_{12}O_5$	175.0619	4.1	8.18	175.0599,130.9661,115.0408,86.9793,81.9547		√
M43	$C_7H_{12}O_6$	191.0565	1.9	3.1	145.0611,99.0087,93.0371,85.0319		√
M44	$C_{15}H_{14}O_6$	289.073	4.3	32.28	414.1994,413.2009,329.1410,255.0799,96.9618,79.9595	√	√
M45	$C_{17}H_{14}O_7$	329.0653	-4.1	44.03	313.0343,329.0651,314.0411,299.0176,271.0239,61.9922	√	
M46	$C_{15}H_{12}O_{12}P_2$	444.971	-4.8	49.81	380.9761,365.2389,330.8826,168.9931,118.9949	√	√
M47	$C_{22}H_{20}O_{16}S$	571.0376	-4	29.31	572.0356,571.0345,491.0772,315.0489,300.0266	√	√
M48	$C_{22}H_{24}O_{10}$	447.1318	4.8	48.17	447.1318,149.0018	√	√
M49	$C_{22}H_{22}O_{12}$	477.1018	-4.2	31.79	477.0989,301.0706,151.0043	√	√
M50	$C_{19}H_{16}O_7$	355.0808	-4.4	42.96	355.0817,297.0401,285.0392,253.0548,133.0314,61.9932	√	
M51	$C_{19}H_{16}O_{14}$	467.0489	4.6	8.5	467.1255,399.0603,263.0875,240.9798,172.9926	√	√
M52	$C_{21}H_{20}O_{18}P_2$	621.0071	3	2.65	553.0187,485.0304,417.0441,349.0574,213.0853	√	√
M53	$C_{18}H_{16}O_7$	343.0837	4	34.42	370.9871,343.0841,219.1387	√	√
M54	$C_{22}H_{24}O_{14}$	511.1112	3.7	2.64	513.1181,512.1143,511.1112,510.8786,506.9699	√	√
M55	$C_{23}H_{24}O_{12}$	491.1174	-4.3	45.37	485.8820,483.0559,482.0613,481.0578,477.2318,476.1025	√	√
M56	$C_{21}H_{18}O_{14}S$	527.047	-3.9	30.73	527.0456，351.0157，271.0591	√	

M57	$C_{15}H_{10}O_{11}S_2$	430.9727	-2.3	33.97	351.0162，271.0588，270.2143，153.0187	√	
M58	$C_{22}H_{20}O_{11}$	461.1062	-3.6	39.77	461.1072，285.0741，270.0524，242.0567	√	
M59	$C_{22}H_{22}O_{10}$	447.127	-3.5	45.38	447.2388，429.1169，385.0918，237.0738	√	√
M60	$C_{16}H_8O_7$	313.0351	2.7	2.6	313.0351	√	√
M61	$C_{22}H_{20}O_9$	429.1168	-2.8	45.37	429.3348,429.2580,429.1207,341.0639,193.0470	√	√
M62	$C_{21}H_{18}O_{11}$	447.0908	-3.2	30.73	447.0913,271.0586	√	
M63	$C_{22}H_{20}O_{12}$	477.1007	-4.2	33.14	477.2434,477.1026,301.0698,286.0464	√	
M64	$C_{16}H_{12}O_{10}S_2$	428.9959	3.4	2.91	428.9959		√
M65	$C_{21}H_{20}O_7$	385.1272	-2.5	5.2	385.2180,385.1344,250.0750,210.0520,204.0380,136.0612,134.0260	√	√
M66	$C_{21}H_{20}O_{11}$	449.1062	-3.6	31.3	449.1057,287.0532,273.0743,153.0176	√	
M67	$C_4H_8O_7$	169.0351	4.8	4.53	169.0333,152.0062,141.0386,126.0284	√	√
M68	$C_6H_{12}O_9S$	261.0277	0.9	4.09	261.0277	√	√
M69	$C_5H_{10}O_5$	151.0607	4	7.91	522.5348,155.1026,151.0575,119.0370	√	√
M70	$C_6H_6O_8$	207.0135	0	2.9	207.0485,142.7816,138.9043	√	√
M71	$C_{11}H_{18}N_2O_8$	307.1149	4.2	16.27	307.1478,307.1139,306.9619,306.1484,211.8609,149.0579	√	
M72	$C_8H_{13}NO_6$	220.0823	3.3	3.25	220.1023,190.1044,174.1096		√
M73	$C_{21}H_{19}O_{15}P$	543.0522	-2.3	28.15	543.2390,543.0538,463.0965,367.0218,287.0657,221.0023	√	
M74	$C_{27}H_{28}N_2O_{14}$	605.1633	3.3	8.38	695.1988,605.1582,431.1818,227.0774,210.0556		√
M75	$C_{17}H_{15}NO_5$	314.1015	-2.5	31.52	250.0832,232.0737,149.0213,120.0793	√	√
M76	$C_{12}H_6O_7$	263.0198	4.2	4.01	263.1761,218.9941,217.0597,174.9703,158.9375,132.1002,116.9620,114.9459	√	√
M77	$C_{16}H_{18}O_4$	275.1269	-3.1	43.82	275.1585,275.1253,234.1138,179.0665,162.0659,133.0636	√	√
M78	$C_{26}H_{29}N_3O_{10}$	544.1916	-1.8	50.93	545.5084,544.3345,526.3241,184.0711,124.9983,104.1057		√
M79	$C_{16}H_{14}O_{10}$	367.0659	-0.1	4.01	367.0659		√
				**Organic acid and their analogues**			
M80	$C_6H_{11}NO_6S$	224.0233	-0.7	3.37	166.0179,124.0084,79.9598	√	√
M81	$C_7H_8O_7$	203.0204	3.1	2.91	203.0152,151.5283,145.0634,135.0320,127.0528,128.0357,75.0109	√	√
M82	$C_9H_{12}O_{10}$	279.0351	-2.4	4.38	282.9363,280.0379,278.9980,279.0351,276.9947,275.0164	√	√
M83	$C_6H_9NO_5$	174.0412	2.1	3.59	174.0406,130.0525,89.0257,88.0420,61.9918	√	√
M84	$C_{12}H_{14}N_2O_4$	249.0877	-1.4	8.2	186.0583,159.0938,142.0684,116.0521,112.9866,96.9622,74.0262	√	
M85	$C_{13}H_{16}O_7$	283.0815	-2.8	14.48	283.0807,186.9317,146.9659,113.0261,107.0521,	√	

M86	$C_9H_9NO_3$	178.0515	3	12.14	178.0515,134.0624,77.0430,61.9922	√	
M87	$C_9H_{11}NO_6S$	260.0233	-0.6	3.96	265.0426,265.0103,261.0062,261.0290,260.0233,257.1494,257.0745,255.9841	√	
M88	$C_{10}H_{11}NO_6S$	272.023	-1.5	5.72	124.0085,79.9595,61.9918	√	
M89	$C_{11}H_{11}NO_3$	204.0668	0.9	21.68	205.0695,204.0653,158.0618,128.0517,116.0521	√	√
M90	$C_{16}H_{20}O_6$	307.118	-2.3	45.35	945.0598,307.1185,263.1621,263.1284,102.9588	√	√
M91	$C_{16}H_{20}O_5$	291.1229	-3	43.82	291.1238,273.1037,247.1357,203.1443,135.0812,96.9632	√	√
M92	$C_{15}H_{16}O_{12}S$	419.0273	-4.1	58.66	421.0249,419.2911,418.0331,417.0295,415.0331	√	√
M93	$C_9H_8O_8S$	274.9875	2.8	3.01	278.8298,276.9854,272.9912,270.7967	√	√
M94	$C_7H_{14}O_9$	241.056	-2.1	8.16	150.9174,145.0508,142.0677,116.0517,101.0611,74.0250	√	√
M95	$C_9H_{10}O_7$	229.0365	4.9	4.04	167.0323,103.0420,59.0179	√	√
M96	$C_7H_{14}O_8$	225.0615	-0.3	2.81	228.9027,226.8166,222.8681,220.8710	√	√
M97	$C_{15}H_{18}O_9$	341.0861	-4.9	15.05	341.0918,267.0047,187.0085,146.9661,119.9497,61.9924	√	√
M98	$C_8H_{14}O_4$	173.0826	3.6	20.11	173.0837,136.9379,129.0934,111.0828,109.0671	√	√
M99	$C_9H_{10}O_3$	165.0564	4.3	14.93	147.0456,119.0524,103.0574,96.9619	√	√
M100	$C_9H_8O_6S$	242.9966	-1.3	21.41	242.9955,163.0405,119.0518	√	
M101	$C_7H_{12}O_8S$	255.0173	-2.8	4.53	222.9904,175.0236,143.0355,117.0226,79.9595	√	
M102	$C_{15}H_{16}O_{10}$	355.0655	-4.4	3.04	191.0517,188.0363,188.0201,145.9990,92.9304,88.0438	√	√
M103	$C_9H_6O_5$	193.0137	-3	3.11	157.0373,140.0109,114.0323,97.0063	√	√
M104	$C_9H_{10}O_4$	181.0512	2.9	9.53	181.0516,180.9287,163.0410,135.0463,112.9869,119.0520,92.9215	√	√
M105	$C_7H_{10}O_6$	189.0408	1.6	4.2	189.0403,129.0198,99.0198,86.0033,71.0167	√	√
M106	$C_9H_{15}NO_5$	216.0879	0.6	3.54	198.0429,180.0660,172.0993,146.0373,128.0748,127.8725,84.0845	√	√
M107	$C_{25}H_{24}O_{16}$	579.0981	-1.9	2.64	580.1006,579.0981,578.8663		√
M108	$C_9H_8O_2$	147.0459	4.8	4.99	204.0670,186.0570,158.0612,142.0680,130.0674,116.0524	√	√
M109	$C_9H_8O_7S$	258.9914	-1.5	18.8	261.8776,259.9945,258.9913255.9212	√	√
M110	$C_{15}H_{18}O_6$	295.1163	-4.3	49.02	107.0479	√	√
M111	C_8H_6O	119.049	-1.6	4.14	119.0646,101.0376,101.0231	√	√
M112	C_9H_{10}	119.0855	0	48.99	119.0855	√	√
M113	$C_{22}H_{26}O_{17}$	563.1249	1.1	2.6	563.1249	√	√
M114	$C_{16}H_{19}O_{10}P$	403.0804	3.9	31.07	153.0152	√	
M115	$C_8H_4O_3$	149.0231	-1.4	45.23	149.0189	√	√

M116	$C_{21}H_{25}NO_{12}S$	516.1152	-3.5	28.15	516.1128，383.1107，267.1025，243.0999	√	√
M117	$C_9H_6O_2$	147.0436	-3	4.14	119.0496	√	√
M118	C_9H_6O	131.0486	-4.5	5.01	103.0517	√	√
M119	$C_9H_8O_3$	165.054	-3.6	3.77	164.9188，147.0476，123.0432，120.0551，119.0495，103.0518	√	
M120	$C_{16}H_{18}O_{12}$	403.0862	-2.2	12.2	591.2265，403.1196，369.0250，289.0675，163.0369	√	
M121	$C_{21}H_{26}N_2O_{11}$	483.1627	3.7	3.2	483.1627	√	√
M122	$C_{26}H_{26}O_{10}$	499.1596	-0.5	48.98	439.2089，437.1928，319.0803，259.0641，124.9889		√
M123	$C_7H_{10}O_7$	207.0499	-0.1	2.69	207.0454，207.0262，142.7816，138.9044	√	√

Table S4. Degree values of 14 chemical components in a multivariate network.

Name	Degree value
Luteolin	13
Diosmetin	5
Hesperetin-7-O-glucuronside	4
Eriodictyol 7-glucuronide	4
Luteolin-7-O-glucoside	3
Luteolin-7-O-glucuronside	3
Apigenin-7-O-glucoside	3
Acacetin	3
Gentisic acid	2
Quercetin-3-O-glucoside	2
Succinic acid	1
Diosmetin-7-O-glucuronside	1
Apigenin-7-O-glucuronside	1
4,5-dicaffeoylquinic acid	1

Table S5. Changes of SBP, MBP and DBP of rats in each group before and after modeling.

Group	SBP (mmHg)			MBP (mmHg)			DBP (mmHg)		
	Day 0	Day 28	Day 28-Day 0	Day 0	Day 28	Day 28-Day 0	Day 0	Day 28	Day 28-Day 0
C	131.5±8.44	116.9±9.75	-14.58±12.85	105.2±7.659	99.5±8.50	-5.625±10.35	91.9±10.97	90.8±8.31	-1.125±13.61
M	130.0±7.878	174.6±19.34	44.60±16.42##	105.6±8.62	146.9±20.36	41.31±16.39##	93.1±10.84	132.9±20.84	39.83±17.75##
CMFL	126.0±6.807	156.5±11.08	30.46±8.31*	107.9±6.779	130.5±12.54	22.58±8.78*	98.7±7.358	117.5±14.06	18.75±11.14*
CMFM	126. 9±4.902	155.3±8.23	28.46±8.52*	108.1±3.878	129.1±7.792	21.00±5.632**	98.4±5.151	115.7±8.89	17.29±5.769**
CMFH	124.8±5.185	147.5±5.188	22.75±4.720**	108.2±3.660	119.4±11.53	11.25±10.22**	99.4±4.929	105.5±16.31	6.167±13.86**
CIFL	130.0±6.489	153.5±9.35	23.50±6.032**	108.8±5.101	132.1±10.62	23.38±8.95*	98.3±4.966	121.4±11.46	23.13±10.86*
CIFM	129.0±4.762	150.0±12.54	20.96±15.11**	111.5±2.569	129.8±12.62	18.33±11.52**	102.4±3.946	119.8±13.94	17.38±12.01*
CIFH	129.8±6.886	149.9±16.77	20.13±17.62*	111.0±5.470	122.4±20.11	11.46±18.45**	101.5±5.164	108.6±23.26	7.083±20.70**
P	137.6±11.78	155.8±13.80	18.25±12.60**	113.2±8.49	127.5±8.72	14.29±8.03**	100.8±9.96	113.1±9.08	12.29±7.606**

Values shown are means±SD. #*P*< 0.05, ##*P*< 0.01 compared with C group; **P*<0.05, ***P*< 0.01 compared with M group.

Table S6. Binding energy results of active components with relevant receptor proteins.

Components	Binding energy to residues of 6OS2 (kcal/mol)				Binding energy to residues of 1O86 (kcal/mol)				
	**ALA1244**	**GLN1257**	**ASN1294**	**ILE1245**	**LYS511**	**TYR520**	**GLN281**	**HIS353**	**GLU384**
Lisinopril-Control Drug of ACEI	——	——	——	——	-4.99	-4.99	-4.99		
Captopril-Control Drug of ACEI	——	——	——	——		-6.84	-6.84	-6.84	-6.84
Losartan-Control Drug of ARBs	-6.83	-5.94	-5.9		——	——	——	——	——
Valsartan-Control Drug of ARBs				-6.8	——	——	——	——	——
Acacetin		-5.74	-5.61			-6.58	-6.58		
Apigenin-7-O-glucoside			-4.42					-7.2	
Diosmetin		-5.04		-5.49	-7.09	-7.09			
Eriodictyol-7-glucuronide	-4.65		-4.22		-7.46	-7.46	-7.46	-7.46	
Hesperetin-7-O-glucuronside	-5.34		-4.07					-8.18	-8.18
Luteolin				-5.12	-7.2	-7.2	-7.2		
Luteolin-7-O-glucoside	-5.02				-7.84	-7.84			
Luteolin-7-O-glucuronside			-4.18	-6.43		-6.83	-6.83		

6OS2-Angiotensin II type 1 receptor crystal structure; 1O86-Angiotensin converting enzyme crystal structure.

Table S7. The analytical performance of plasma samples in positive mode.

m/z- t_R (min)	Precision (RSD %)		Repeatability (RSD %)		Stability (RSD %)	
	t_R	Intensity	t_R	Intensity	t_R	Intensity
147.1-2.1	0.0	6.3	0.1	4.6	0.1	3.5
349.1-4.3	0.1	5.0	0.1	2.0	0.1	5.5
201.1-7.2	0.1	2.4	0.1	2.2	0.1	5.0
836.4-12.0	0.1	4.6	0.1	5.8	0.0	5.3
457.3-23.0	0.1	5.7	0.1	4.7	0.1	3.9
408.3-32.0	0.1	8.3	0.1	5.6	0.0	8.2

Table S8. The analytical performance of plasma samples in negative mode.

m/z-t_R (min)	Precision (RSD %)		Repeatability (RSD %)		Stability (RSD %)	
	t_R	Intensity	t_R	Intensity	t_R	Intensity
344.9-2.0	0.6	4.6	0.7	3.6	0.7	4.9
581.3-11.7	0.2	2.2	0.1	3.6	0.3	4.1
527.3-19.2	0.0	9.3	0.1	2.7	0.1	5.2
558.3-25.5	0.1	6.5	0.4	6.8	0.1	4.0
395.2-29.2	0.2	8.8	0.2	6.5	0.2	6.6
446.9-34.8	0.0	5.5	0.1	8.0	0.1	4.2

Table S9. Typical biomarkers information and the callback effect of drugs.

No.	*m*/*z*	t_R (min)	Name	M group compared to C group				Callback effect compared to M group		
				P	VIP	FC	Trend	CMF	CIF	P
1	603.4388	26.68	(5Z,8Z,11Z,14Z,17Z)-Icosapentaenoic acid	1.17E-03	1.78	3.80	↓			
2	299.25859	24.98	(R)-10-Hydroxystearate	7.30E-04	1.83	2.13	↓			
3	134.0188	2.80	(S)-Malate	4.14E-03	1.70	4.47	↓	√ ■		√
4	315.19569	16.33	15-Deoxy-Delta12,14-PGJ2	3.60E-04	1.88	2.29	↓	√ ■		
5	271.22751	22.34	16-Hydroxypalmitate	1.51E-03	1.74	2.43	↓			
6	320.23035	23.83	20-HETE	2.64E-02	1.37	4.04	↓			
7	189.00418	11.54	2-Hydroxyphenylacetate	5.83E-08	2.25	4.09	↓	√	√ ▲	
8	405.26287	16.95	3alpha,7alpha-Dihydroxy-12-oxo-5beta-cholanate	1.83E-02	1.40	3.13	↓			
9	284.08513	11.32	4-Amino-4-deoxychorismate	1.53E-06	2.19	4.20	↓		√ ▲	
10	317.21141	22.74	5(S)-HPETE	2.46E-02	1.36	8.11	↓			
11	468.30407	32.95	Cholesterol sulfate	1.41E-02	1.60	0.40	↑	√		
12	406.03031	3.28	Citrate	8.55E-03	1.65	2.10	↓			√
13	243.04856	3.62	Cystathionine	1.70E-02	1.55	2.21	↓			√
14	333.20601	16.57	Delta-12-Prostaglandin J2	3.52E-03	1.65	3.18	↓			
15	352.07165	11.32	Dihydropteroate	2.47E-06	2.17	3.95	↓		√ ▲	
16	318.21496	22.76	8(R)-HPETE	2.93E-02	1.33	8.59	↓			
17	377.1491	14.93	Methyl (indol-3-yl)acetate	1.91E-02	1.40	3.43	↓	√	√	√
18	407.12088	11.03	Phenylacetylglycine	1.39E-03	1.77	2.55	↓			
19	315.23186	27.41	Pregnenolone	2.34E-03	1.72	2.19	↓			
20	498.28666	16.39	Taurochenodeoxycholate	4.91E-02	1.27	2.05	↓			
21	187.00741	11.54	Terephthalate	6.48E-08	2.25	6.34	↓		√ ▲	√
22	320.25791	26.70	(5Z,7E,9E,14Z,17Z)-Eicosapentaenoic acid	2.46E-02	1.58	2.07	↓			
23	343.22399	23.86	14,15-EET	2.53E-02	1.27	4.21	↓			
24	359.19126	23.88	8,9-EET	2.21E-02	1.30	3.81	↓			
25	408.3103	20.23	3alpha,12alpha-Dihydroxy-5beta-chol-6-enoate	7.30E-04	1.67	2.08	↓			

26	357.2784	19.87	3-Oxo-5beta-cholanate	2.76E-03	1.56	2.14	↓			
27	375.28874	19.86	Deoxycholic acid	4.69E-03	1.48	2.52	↓			
28	268.10371	6.65	Adenosine	4.36E-03	1.67	0.41	↑	√ ■	√	
29	348.28659	26.71	Anandamide	9.89E-03	1.61	8.72	↓	√	√	
30	787.60084	19.17	Chenodeoxycholate	3.60E-03	1.57	3.37	↓	√	√	√
31	809.58536	25.73	Hyodeoxycholate	4.66E-02	1.29	2.10	↓	√	√	√
32	786.59812	21.07	Murideoxycholic acid	2.75E-03	1.73	3.98	↓	√	√	√
33	391.28365	20.19	Cholic acid	4.10E-03	1.57	2.16	↓			
34	477.169	3.24	Deoxycytidine	8.60E-07	2.19	0.37	↑			√
35	158.09253	3.40	L-Citrulline	3.65E-03	1.62	2.57	↓			
36	341.20854	22.78	Leukotriene A4	2.30E-02	1.33	6.10	↓			
37	301.21618	22.78	γ-linolenic acid	2.46E-02	1.28	6.95	↓			
38	839.63195	32.78	L-Palmitoylcarnitine	9.38E-07	2.13	3.40	↓	√ ■	√	√
39	175.04729	8.52	N1-Methyl-4-pyridone-5-carboxamide	4.44E-02	1.33	0.10	↑			
40	782.56528	26.92	Sphinganine 1-phosphate	3.24E-02	1.44	2.01	↓	√	√	
41	139.0498	3.62	Urocanate	4.56E-05	1.98	2.22	↓	√ ■	√	√

▲*P*<0.05, compared with CMF group, and the effect of CIF was significantly better than that of CMF; ■*P*<0.05, compared with CIF group, and the effect of CMF was significantly better than that of CIF.

Supplementary Figures

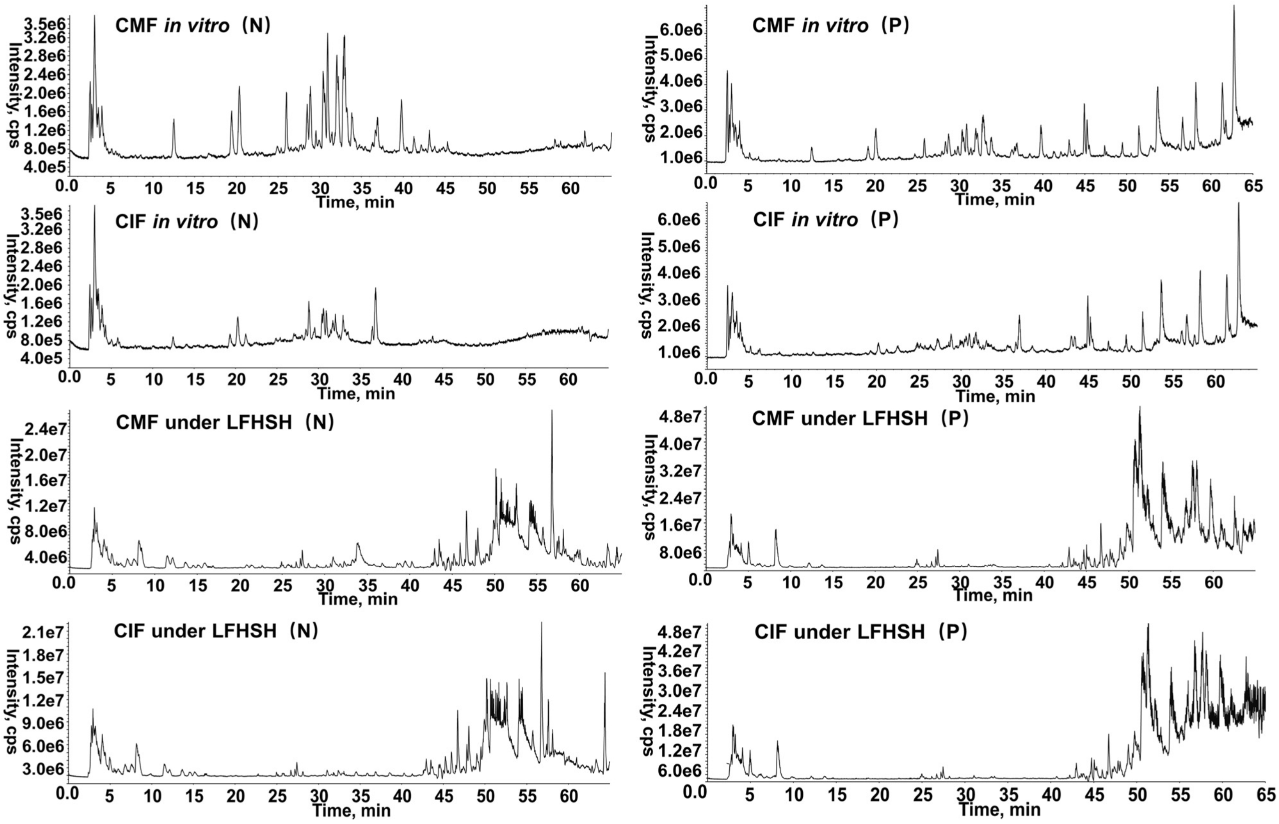

Figure S1. The total ion chromatograms for identification of *in vivo* and *in vitro* components of CMF and CIF in positive (P) and negative (N) ion modes.

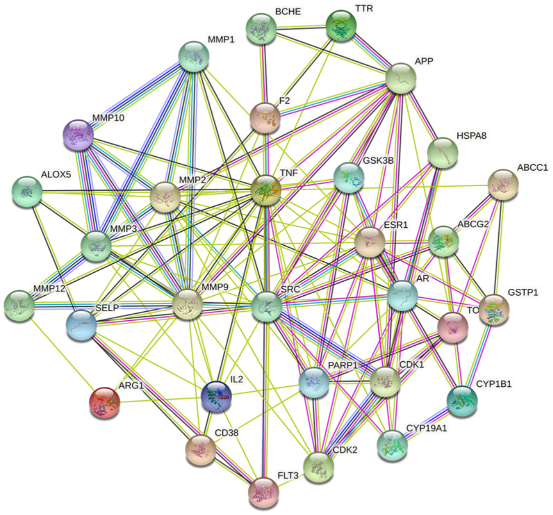

Figure S2. The protein interaction network diagram of 31 potential targets of CMF and CIF in the prevention of LFHSH.

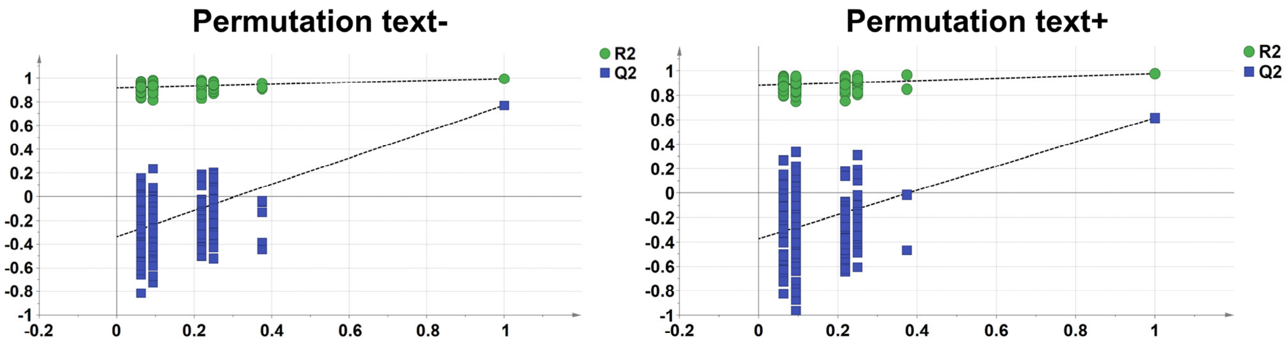

Figure S3. Permutation tests of OPLS DA in negative and positive mode.
